# Lipomatous hypertrophy of the interatrial septum: Risk factors, correlation with thoracic fat composition and clinical impact

**DOI:** 10.1016/j.ejro.2026.100801

**Published:** 2026-07-28

**Authors:** Stefanie Meiler, Quirin Strotzer, Lucca Scheuermeyer, Luca Kronast, Simone Hammer, Christian Stroszczynski, Okka Wilkea Hamer

**Affiliations:** a Department of Radiology, Regensburg University Medical Center, Regensburg, Germany; b Department of Radiology, Kliniken Suedostbayern Traunstein, Traunstein, Germany; c Department of Radiology, Caritas Hospital St. Maria Donaustauf, Donaustauf, Germany

**Keywords:** Lipomatous Hypertrophy, Interatrial Septum, Fat, Risk Factors, Segmentation

## Abstract

**Introduction:**

Lipomatous Hypertrophy of the interatrial septum (LHIS) is a benign, non-encapsulated fat deposition in the septum secundum and is typically reported at a minimum thickness of 1–2 cm. A large-scale volumetric analysis of LHIS has not yet been conducted. Moreover, the roles of risk factors, thoracic fat composition, and clinical significance are still unclear.

**Methods:**

473 adults with interatrial septal thickness ≥ 1 cm on thoracic CT (March 2021–March 2022) were included. Segmentation was performed with a custom Python pipeline and 3D nnU-Net algorithm to quantify septal volume and thoracic fat compartments. Correlations with demographics, metabolic disorders, comorbidities, noxes, and steroid use were analyzed.

**Results:**

LHIS volume strongly correlated with diameter. Age (p = 0.009) and maximum LHIS diameter (p = 0.025) were positively associated with AF (Atrial Fibrillation). Steroid (p = 0.018) use and epicardial fat volume (p < 0.001) significantly influenced LHIS size. Epicardial (EFT) and mediastinal fat (MFT) correlated with female sex, age, BMI (Body Mass Index) and steroid use (p < 0.001, respectively). When grouping patients according to LHIS severity, higher-grade LHIS was linked to BMI, EFT, MFT, smoking, tumor, AF, and steroid use.

**Discussion/Conclusion:**

The study results suggest that a non-anatomical, strictly axial measurement of the extent of the interatrial septum is a reliable surrogate marker for its volume. The presence and the severity of LHIS are predictive of atrial fibrillation. There are various distinct factors influencing the extent of maximum LHIS diameter and volume, as well as epicardial and mediastinal fat tissue.

## 1 Introduction

Lipomatous hypertrophy of the interatrial septum (LHIS) is characterized by a localized non-encapsulated fatty infiltration of the interatrial septum. The fat deposition affects the embryologic septum secundum whereas the fossa ovalis being the remnant of the foramen ovale derived from the thin septum primum is spared. This leads to the typical “dumbbell” shape of the thickened septum on imaging. The condition was first identified during an autopsy by Prior in 1964 [1], but it was not detected in living patients until 1982, following advancements in cardiac imaging techniques [2]. In literature thresholds ranging between 1 and 2 cm are reported regarding the minimum short axis thickness of the interatrial septum (IAS) at which LHIS is to be reported. The most established value is 2 cm [2], [3], [4], [5], [6], [7], [8], [9], [10], [11].

The prevalence of LHIS varies based on the diagnostic method employed: it is observed in approximately 1% of autopsy cases, up to 2.2% in computed tomography (CT), and as much as 8% in transthoracic echocardiography (TEE) studies [12], [13], [14]. Some studies have linked LHIS with obesity and advanced age [4], [10]. Data about clinical manifestation of LHIS remains conflicting. It has been linked to symptoms such as arrhythmias, including atrial fibrillation or supraventricular tachycardia, as well as palpitations, syncope, or even signs of congestive heart failure [11].

Most of the available literature on this topic is limited to case reports [2], [3], [5], [7], [8], [9], [12], [15], [16], [17], [18], [19] and reviews [4], [13] with only a few original research studies published [10], [20], [21]. These studies primarily focus on the diameter, and occasionally the area, of lipomatous hypertrophy. A recent study from Italy explored the extent of LHIS and its relationship with abdominal and subcutaneous fat [20].

To the best of our knowledge, neither a volumetric analysis of the LHIS in a large cohort nor an assessment of its clinical significance has been performed so far. The goal of this study is to contribute to a more comprehensive knowledge of risk factors, correlation with thoracic fat composition and clinical impact.

## 2 Methods

This retrospective study was approved by the institutional ethics committee (21–2718–101). Written informed consent was waived. All procedures were performed in compliance with the ethical standards outlined by the institutional and/or national research committee, as well as the 1964 Helsinki Declaration and its later revisions or comparable ethical principles.

### 2.1 Patient data

A comprehensive database search of all CT scans was performed using the Radiological Information System (RIS, Nexus.medRIS, Version 8.42, Nexus, Villingen-Schwenningen, Germany). The query included the terms “lipomatous hypertrophy of the (inter)atrial septum,” “lipomatous septal hypertrophy,” or “lipomatous hypertrophy” in combination with “(inter)atrial septum”. In this way, 94 patients were identified. Additionally, all thoracic CT scans conducted at the time of data collection from March 2021 until March 2022 were manually reviewed to identify cases of this pathology, even if the radiological reports did not explicitly include these keywords. Using this approach, another 379 patients were detected.

Adult patients (aged 18 years or older) with an interatrial septum diameter (ISD) of minimum 1 cm on axial CT imaging were eligible for inclusion. For patients with multiple scans, only the most recent CT was analyzed. Exclusion criteria included patients under 18 years of age, septal thickness below 1 cm, or the absence of CT imaging in cases where only MRI was available.

Patient data, including demographic information (age, gender, height, weight, and substance use such as alcohol and nicotine), medication history (including steroid use within the last two years), and comorbidities (malignant tumor, arterial hypertension, diabetes, cardiac arrhythmias and coronary artery disease), were extracted from electronic medical records. In the case of nicotine use, alcohol intake, and steroid therapy, the presence of these factors was noted; however, the amount consumed or the dosage was not assessed.

### 2.2 CT images

The imaging studies were performed at two facilities: a tertiary care center (347 out of 473 scans, 73.4%) and a secondary care hospital specializing in pulmonary diseases (126 out of 473 scans, 26.6%). Scans were conducted using Siemens Healthcare CT scanners (Somatom Definition Flash with 128 slices, Somatom Definition AS with 128 slices, or Somatom Go Top with 64 slices). The tube voltage was automatically determined, with a reference voltage of 120 kV, and tube current was modulated according to patient requirements, with reference mAs values ranging from 20 to 352. Images were reconstructed in the axial plane with a 1 mm slice thickness and an increment of 0.7–1 mm, using lung and soft tissue kernels.

Of all CT scans, 87.7% (415 out of 473) were contrast-enhanced, while 12.3% (58 out of 473) were performed without contrast. Among the enhanced scans, most were in the arterial phase (392 out of 415, 94.5%), followed by the pulmonary arterial phase (21 out of 415, 5%) and the venous phase (2 out of 415, 0.5%). Contrast agent Iohexol (Accupaque 350, GE Healthcare) was administered in a weight-adjusted manner, up to a maximum dose of 70 ml, at a flow rate of either 3 ml/s or 4 ml/s. All images were stored in the picture archiving and communication system (PACS) using Syngo Imaging (Siemens, Erlangen, Germany) and Sectra (Sectra AB, Linköping, Sweden).

### 2.3 CT image analysis

Axial chest CT scans in soft tissue kernel were exported in Medicine (DICOM) format. The data were de-identified and transferred to an on-site Linux Ubuntu computer (version 20.04; Canonical Foundation) for further processing. A custom processing routine was developed in Python (v3.8.12; Python Software Foundation). The CT scans were converted to the Neuroimaging Informatics Technology Initiative (NIFTI) file format and resampled to a section thickness of 5 mm to facilitate manual segmentation corrections.

A subset of 45 scans, comprising both contrast-enhanced and non-enhanced scans, was randomly selected to develop a fully automated segmentation algorithm for relevant mediastinal structures. Manual segmentation of the images of this subgroup was performed by a medical student (A.A.) who was specifically trained beforehand using ImFusion Labels (v0.19.3; ImFusion GmbH, Munich, Germany). Labels were verified and corrected where needed by a fourth-year radiology resident (B.B.). The following different compartments were separately segmented and labeled: superior vena cava (between the upper cavo-atrial junction and the entry of the azygos vein), interatrial septum (limited by the superior and inferior walls of both atria), heart (bounded by the pericardium), and mediastinum (bounded as follows: Superiorly by the thoracic inlet, inferiorly by the diaphragm, anteriorly by the sternum, posteriorly by the thoracic vertebral column (extending up to 1 cm posteriorly to the anterior border of the vertebral bodies) and laterally by the parietal pleura of the lungs). A single fold of the self-configuring 3D nnU-Net (v1) with default parameters was trained over 200 epochs using these manual segmentations [22].

The trained model was then applied to the remaining dataset not used for training. A rule-based post-processing algorithm was implemented to refine the segmentations by retaining only the largest connected components for each label and removing any labeled voxels outside the mediastinum. All segmentations were visually inspected, manually corrected and validated where necessary by C.C. (a medical student specifically trained for this task). Based on these patients, the following performance was calculated: The mean Dice similarity coefficient across all foreground labels was 0.997 ± 0.004. Label-specific values were 0.992 ± 0.017 for the SVC, 0.992 ± 0.024 for the interatrial septum, 0.997 ± 0.008 for the heart, and 0.992 ± 0.012 for the mediastinum. All automatically generated segmentations were manually checked and corrected if necessary. For the purposes of this study, minimal corrections are defined as corrections that only affect a small area of segmentation, mostly smaller than approximately 500 voxels, every correction of a bigger area or cumulative corrections bigger than that are not considered minimal.

From the segmented dataset, key parameters were programmatically extracted, including mediastinal fat volume (ml), epicardial fat volume (ml), atrial septum volume (ml), and the maximum ISD in axial sections (mm). For fat volume quantification, a HU-based threshold (−30 and −190 HU) was applied, following Wheeler et al. [23].

The automatic segmentation was performed primarily for reasons of time efficiency and practical feasibility and was not a subject of the present study.

### 2.4 Statistical analysis

Continuous variables are expressed as the mean (standard deviation), ordinal variables as the median (range), and categorical variables as counts with corresponding percentages. The binary coding was applied as follows: 1 = positive, 0 = negative; for the variable sex, 1 = male, 0 = female.

Statistical analyses have been implemented using the Python (v. 3.11) libraries numpy (v. 2.2.0, https://numpy.org), pandas (v. 2.2.3, https://pandas.pydata.org), scipy (v. 1.14.1, https://scipy.org) and statsmodels (v. 0.14.4, https://www.statsmodels.org). Matplotlib (v. 3.9.0, https://matplotlib.org) was used for visualization.

Multiple linear regression models were calculated using the statsmodels (v. 0.14.4, https://www.statsmodels.org/stable/index.html) library implemented in Python to quantify the influence each individual patient parameter has on the variables describing the interatrial septum, as well as the patient’s thoracic fat distribution. A logistic regression model with atrial fibrillation as the dependent variable was similarly constructed.

Binomial tests were used for comparison of proportional differences in binary variables between groups, whereas a one-way ANOVA with a post-hoc Tukey’s test was calculated to assess group differences for metric variables. For this purpose, the variable maximum ISD was divided into three groups: Group 1 (ISD < 2 cm), Group 2 (2 cm ≤ ISD < 3 cm) and Group 3 (ISD ≥ 3 cm). These groups were constructed for the purpose of analyzing group differences, specifically grounded in prior definitions provided in preceding research on LHIS.

A p-value < 0.05 was deemed statistically significant. Due to the exploratory nature of this study, no alpha correction was employed.

## 3 Results

### 3.1 Patient collective

A total of 473 patients were included in the study, comprising 72.7% males (344/473) and 27.3% females (129/473). Mean age was 68 years (SD 8.67).

Body mass index (BMI) was available for 423 of these patients, with a mean value of 29.43 kg/m^2^ (SD 6.75).

Information regarding alcohol and nicotine use, steroid medication, and specific (metabolic and cardiac) comorbidities is summarized in Table S1. Multiple types of cardiac arrhythmias were recorded in the patient collective. However, some of these had very low case numbers. Thus, the analysis was limited to those types of arrhythmias which affected at least 10 patients. Indications for CT scans are detailed in Table S2.

### 3.2 CT segmentation

All automatically generated segmentations were manually checked and corrected if necessary. For 5 cases of the whole sample no corrections to the automatic segmentation result were necessary. For 162 cases minimal corrections were applied, for example small corrections to the cardiac contour or to the limits of the upper mediastinum. For the rest of the sample more extensive manual corrections were necessary.

**Fig. 1:**
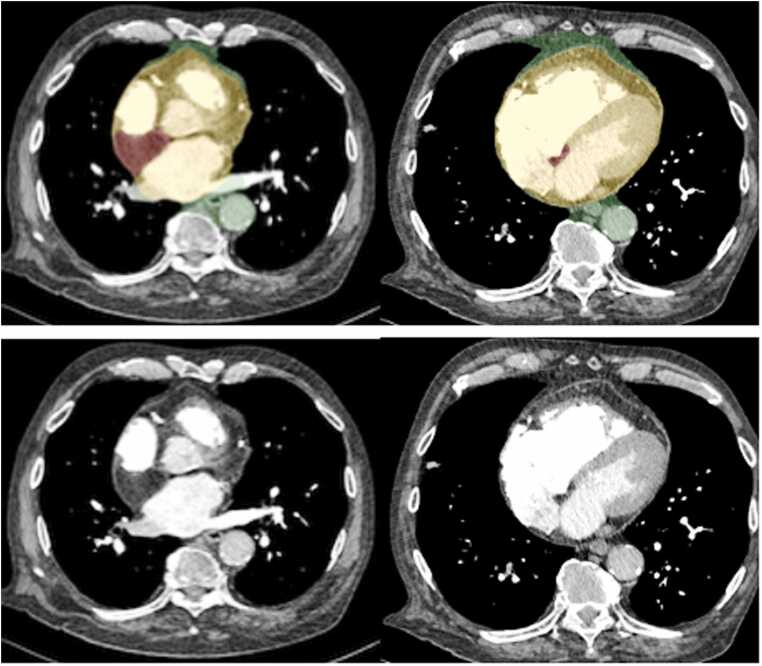
Exemplary case of segmentation in a 74‑year‑old man. Axial CT images at two different levels are shown. The lipomatous hypertrophy of the interatrial septum is highlighted in red, the cardiac structures (including the calculated epicardial fat tissue, EFT) are highlighted in yellow, and the mediastinal fat tissue (MFT) is highlighted in green. Note that a Hounsfield‑unit–based threshold from −190 to −30 HU was applied for fat volume quantification.

In this cohort, the vast majority of thoracic CT examinations were performed with intravenous contrast, and only a small proportion were non-contrast scans. Visual inspection and manual correction of all automated segmentations did not show any relevant differences in segmentation quality between contrast-enhanced and non-enhanced images, indicating that segmentation performance was stable across both types of acquisitions. Similarly, within the contrast-enhanced group, no relevant differences in segmentation performance were observed between arterial, pulmonary arterial, and venous phase scans. As all segmentations were reviewed and corrected, if necessary, the use or timing of contrast did not have a meaningful impact on HU-based fat quantification or on the main study results (Fig. 1).

#### 3.2.1 Interatrial septum

**Fig. 2:**
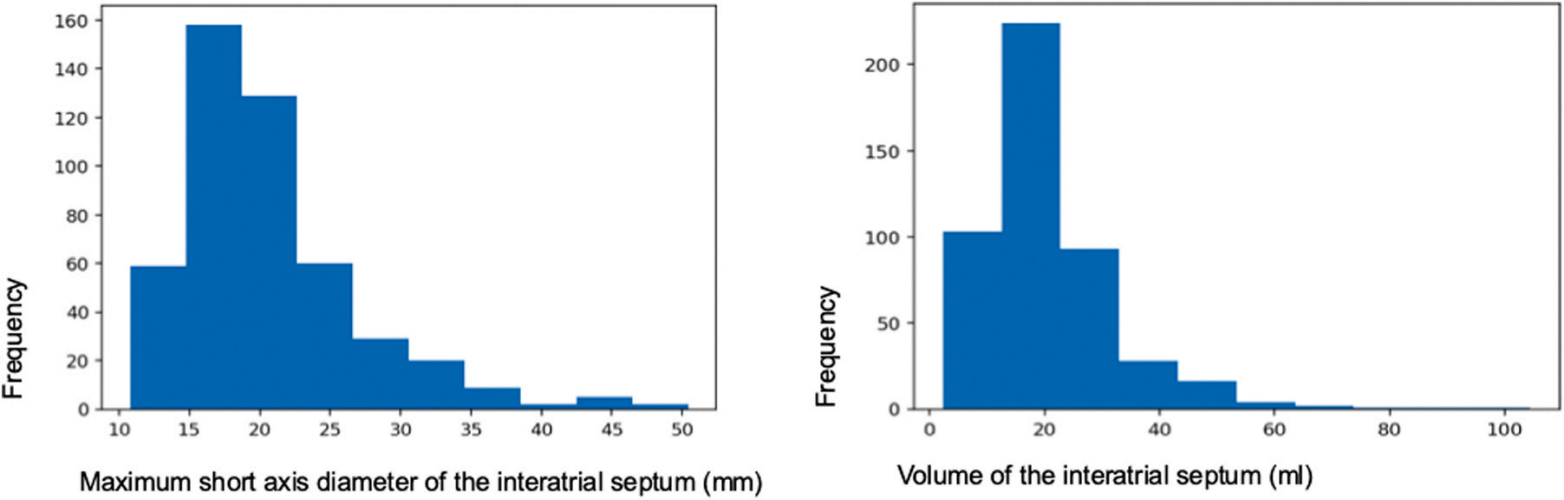
Distribution of the mean maximum ISD (in mm, left) and of the volume (in ml, right) of the interatrial septum (IAS). SD = Standard Deviation.

The mean maximum ISD was 20.57 mm (SD 6.35), and the mean volume was 20.66 ml (SD 11.76). 204/473 (43%) of maximum ISD were greater than 20 mm. Distribution can be seen in Fig. 2.

#### 3.2.2 Fat tissue

**Fig. 3:**
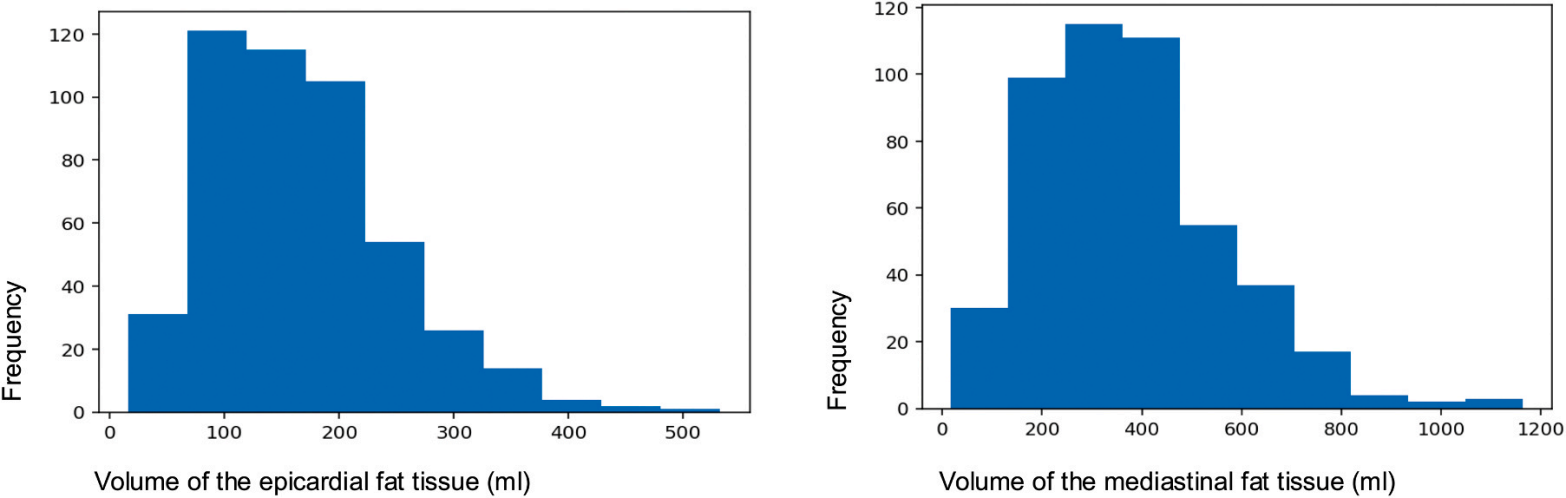
Distribution of the volume of the epicardial fat tissue (in ml, left) and of the volume of the mediastinal fat tissue (in ml, right). SD = Standard Deviation.

The mean volume of the epicardial fat tissue was 168.07 ml (SD 78.85), the mean volume of the mediastinal fat tissue was 374.31 ml (SD 187.30). Distribution is illustrated in Fig. 3.

#### 3.2.3 Relationships between the CT segmentation variables

**Fig. 4:**
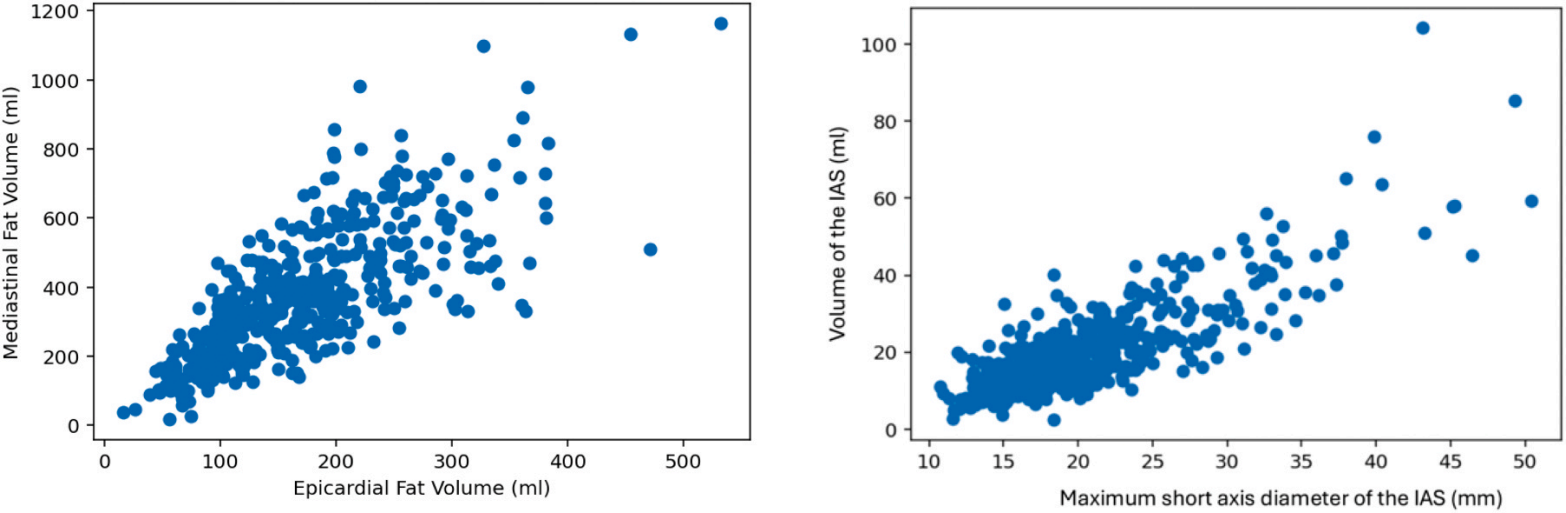
Scatter plot for the relationship between maximum ISD and volume of the interatrial septum (IAS; r = 0.82) and between epicardial and mediastinal fat tissue volume (r = 0.75).

There was a positive linear relationship between the maximum ISD and the volume of the interatrial septum (Pearson’s r = 0.82) as well as between the epicardial and mediastinal fat tissue (Pearson’s r = 0.75, Fig. 4).

### 3.3 Influence of patient characteristics on the interatrial septum and thoracic fat distribution - Multiple linear regression models

Multiple linear regression models including individual patient characteristics (sex, age, BMI, tumor, noxes, metabolic disorders, steroid medication, maximum ISD and volume as well as epicardial/mediastinal fat tissue) were performed for the subgroup with available BMI data (n = 423) to quantify the influence on the measures of the IAS and thoracic fat distribution. Due to a predictably high correlation between the maximum ISD and the volume of the IAS, these parameters were not used as predictors for each other to avoid collinearity. The same applied for epicardial and mediastinal fat volume. Concerning arrhythmias, the group sizes of tachycardia (n = 17) and AV-blocks (n = 13) were much smaller in comparison to AF (n = 83). Therefore, the statistical analysis was limited to the latter. Results can be seen in Table 1a, Table 1b, Table 1c, Table 1d.

**Table 1a:** Multiple linear regression models to analyze the predictive value of individual patient characteristics on the variable *maximum ISD*, R^2^= 0.317. AF = Atrial Fibrillation, EFT = Epicardial Fat Tissue. Non-significant parameters were as follows: age, BMI, tumor, smoking, hypertension, diabetes, CAD.

Variable	coefficient	Standard error	t	P value	95% Confidence
sex	0.2989	0.117	2.565	**0.011**	[0.070, 0.528]
alcohol	0.2574	0.124	2.084	**0.038**	[0.015, 0.500]
AF	0.2557	0.112	2.290	**0.023**	[0.036, 0.475]
steroid	0.2139	0.090	2.370	**0.018**	[0.037, 0.391]
EFT (ml)	0.5159	0.070	7.369	**< 0.001**	[0.378, 0.653]

**Table 1b:** Multiple linear regression models to analyze the predictive value of individual patient characteristics on the variable *volume of the interatrial septum*, R^2^= 0.438. CAD = Coronary Artery Disease, AF = Atrial Fibrillation, EFT = Epicardial Fat Tissue. Non-significant parameters were as follows: sex, age, BMI, tumor, smoking, alcohol, hypertension, diabetes.

Variable	coefficient	Standard error	t	P value	95% Confidence
CAD	0.2547	0.097	2.633	**0.009**	[0.065, 0.445]
AF	0.4576	0.101	4.520	**< 0.001**	[0.259, 0.657]
steroid	0.1779	0.082	2.175	**0.030**	[0.017, 0.339]
EFT (ml)	0.5397	0.063	8.503	**< 0.001**	[0.415, 0.665]

**Table 1c:** Multiple linear regression models to analyze the predictive value of individual patient characteristics on the variable *volume of epicardial fat*, R^2^= 0.351. Non-significant parameters were as follows: smoking, alcohol, hypertension, diabetes, CAD, AF. *Data missing for 50 participants owing to missing values in height and/or weight.

Variable	coefficient	Std. error	t	P value	95% Confidence
sex	−0.4721	0.092	−5.127	**< 0.001**	[−0.653, −0.291]
age	0.0198	0.005	3.971	**< 0.001**	[0.010, 0.030]
BMI*	0.4433	0.042	10.476	**< 0.001**	[0.360, 0.526]
tumor	−0.2381	0.086	−2.778	**0.006**	[−0.407, −0.070]
steroid	0.3585	0.085	4.204	**< 0.001**	[0.191, 0.526]

**Table 1d:** Multiple linear regression models to analyze the predictive value of individual patient characteristics on the variable f *volume of mediastinal fat*, R^2^= 0.466. BMI = Body Mass Index. Non-significant parameters were as follows: tumor, smoking, alcohol, hypertension, CAD, AF. *Data missing for 50 participants owing to missing values in height and/or weight.

Variable	coefficient	Standard error	t	P value	95% Confidence
sex	−1.1189	0.084	−13.394	**< 0.001**	[−1.283, −0.955]
age	0.0164	0.005	3.635	**< 0.001**	[0.008, 0.025]
BMI*	0.4649	0.038	12.111	**< 0.001**	[0.389, 0.540]
diabetes	0.1709	0.085	2.013	**0.045**	[0.004, 0.338]
steroid	0.3681	0.077	4.760	**< 0.001**	[0.216, 0.520]

For the sake of clarity, only the significant variables from the analyses were included in the table.

#### 3.3.1 Interatrial septum

##### 3.3.1.1 Maximum ISD

Regression analyses showed a significant effect of sex (p = 0.011), alcohol consumption (p = 0.038), AF (p = 0.023), steroid medication (p = 0.018) and epicardial fat volume (p < 0.001) on the maximum extent of the short-axis diameter of the IAS. Please see Tables 1a.

##### 3.3.1.2 Volume

For the IAS volume, alcohol consumption and sex were no significant predictors, all others remained significant analogous to the maximum ISD (see above) with addition of CAD (p = 0.009). Please see Table 1b.

#### 3.3.2 Thoracic fat distribution

##### 3.3.2.1 Epicardial fat tissue

Sex (p < 0.001), age (p < 0.001), BMI (p < 0.001), presence of a tumor (p = 0.006), as well as steroid medication (p < 0.001) demonstrated a significant influence on epicardial fat volume. Hereby, the regression revealed significant negative coefficients for the variable tumor on epicardial fat volume. Please see Table 1c.

##### 3.3.2.2 Mediastinal fat tissue

Regarding mediastinal fat volume, the same predictors reached significance as seen with epicardial fat tissue with the exception of the variable malignant tumor and in addition of the variable diabetes (p = 0.045). Please see Table 1d.

### 3.4 Proportional differences between binary variables - Binomial tests

**Fig. 5:**
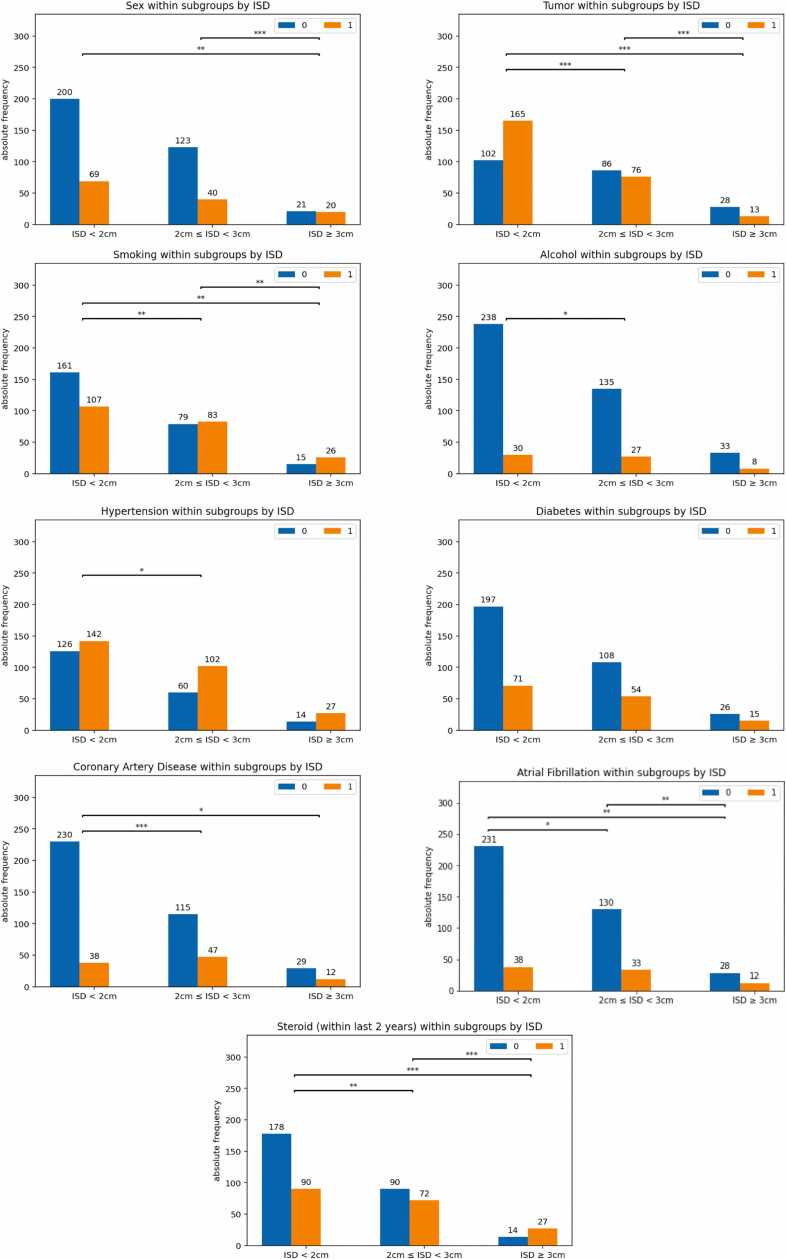
Comparison of the ISD-Subgroups – Sex (1 = male, 0 = female), Tumor, Smoking, Alcohol, Hypertension, Diabetes, Coronary Artery Disease (CAD), Atrial Fibrillation (AF) and Steroid (1 = yes, 0 = no; respectively). ISD = Maximum Interatrial Septum Diameter. * = p < 0.05, ** = p < 0.01, *** = p < 0.001.

Binomial tests were performed to identify proportional differences between binary characteristics. For this purpose, the variable *maximum ISD* was divided into three groups: Group 1 (ISD < 2 cm), Group 2 (2 cm ≤ ISD < 3 cm) and Group 3 (ISD ≥ 3 cm). The patient characteristics sex, malignant tumor, smoking, alcohol, hypertension, diabetes, CAD, AF and steroid were included in the analysis. Please see Table 2, Table S3 and Fig. 5.

**Table 2:** Binomial tests for proportional differences between binary variables. CAD = Coronary Artery Disease, AF = Atrial Fibrillation.

Groups		Group 1 vs. Group 2	Group 2 vs. Group 3	Group 1 vs. Group 3
p-values	sex	0.788	**< 0.001**	**0.002**
	tumor	**< 0.001**	**< 0.001**	**< 0.001**
	smoking	**0.006**	**0.001**	**0.004**
	alcohol	**0.034**	0.375	0.129
	hypertension	**0.018**	0.409	0.118
	diabetes	0.076	0.372	0.16
	CAD	**< 0.001**	1.0	**0.011**
	AF	**0.032**	**0.012**	**0.011**
	steroid	**0.005**	**< 0.001**	**< 0.001**

### 3.5 Proportional differences between metric variables - One-way ANOVAs incl. post-hoc Tukey’s Test

**Fig. 6:**
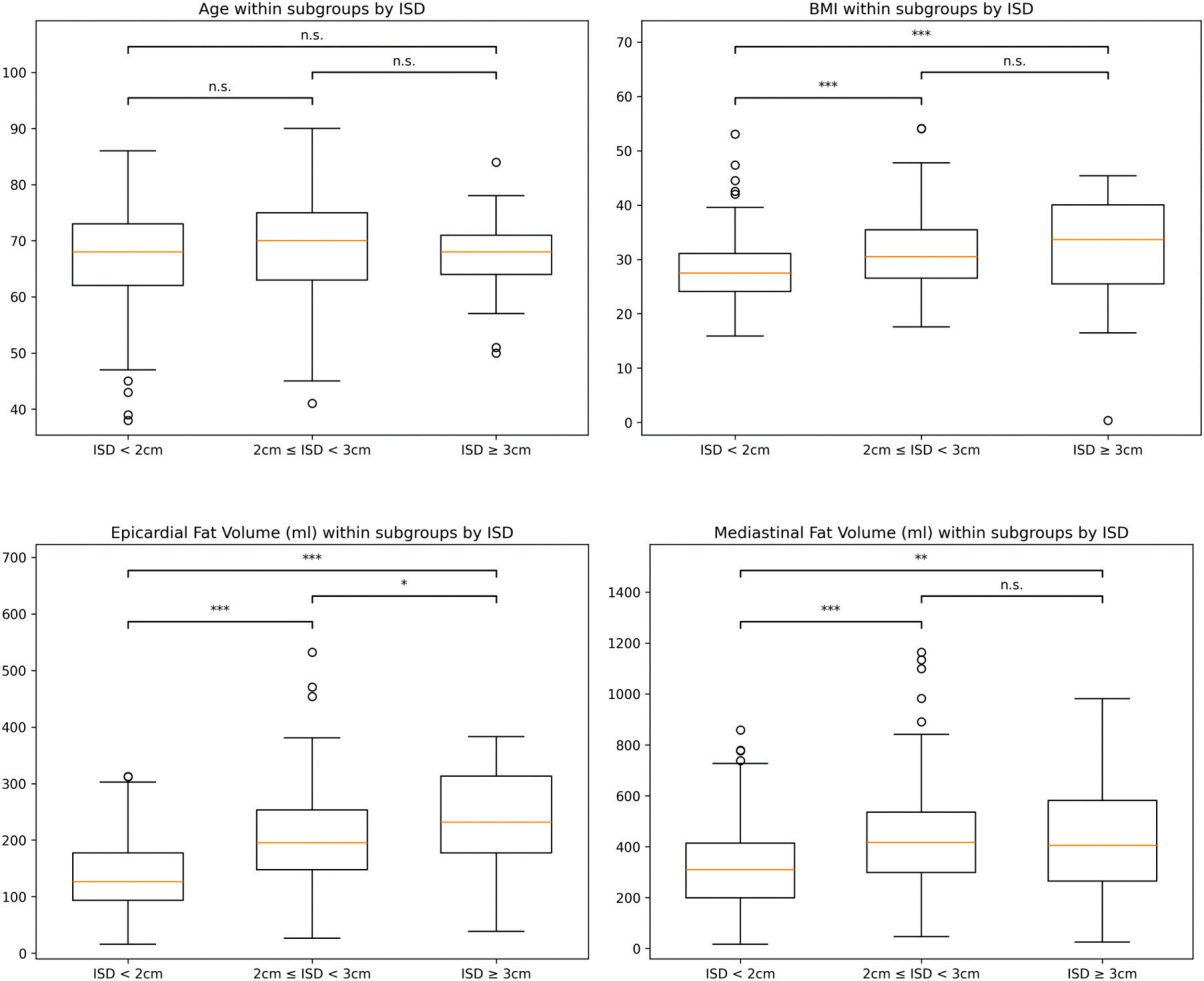
Comparison of the ISD-Subgroups – Age, BMI, Epicardial Fat Volume, Mediastinal Fat Volume. ISD = Maximum Interatrial Septum Diameter, n.s. = not significant. * = p < 0.05, ** = p < 0.01, *** = p < 0.001.

One-way ANOVAs were conducted to assess group differences for metric variables. Corresponding to binomial tests, the variable maximum ISD was categorized into three groups: Group 1 (ISD < 2 cm), Group 2 (2 cm ≤ISD < 3 cm), and Group 3 (ISD ≥ 3 cm). The analysis included the variables age, BMI, as well as the parameters of the thoracic fat tissue and of the interatrial septum. If the F-test was significant, additional post-hoc Tukey’s tests were performed for pairwise group comparisons. Please see Table 3 and Fig. 6.

**Table 3:** One-way ANOVAs incl. post-hoc Tukey’s Test for Proportional differences between metric variables. *not applicable due to ANOVA F-Test: p = 0.076.

variables	ANOVA F-Test p-values	Post-hoc Tukey’s Test p-values		
		Group 1 vs. Group 2	Group 2 vs. Group 3	Group 1 vs. Group 3
age	p = 0.076	n.a.*	n.a.*	n.a.*
BMI	p < 0.001	**< 0.001**	0.91	**0.001**
EFT	p < 0.001	**< 0.001**	**0.017**	**< 0.001**
MFT	p < 0.001	**< 0.001**	0.964	**0.001**

### 3.6 Logistic regression model for atrial fibrillation

To investigate the influence of the observed variables on the probability of AF within the sample, a logistic regression model was estimated. Four variables demonstrated statistical significance: Age, malignant tumor, smoking, and ISD.

Age had a positive coefficient (coef = 0.0476, p = 0.009). Malignant tumor was negatively associated with AF (coef = −0.9365, p = 0.001). Smoking also showed a significant negative association (coef = –0.6447, p = 0.029). Maximum ISD had a positive and significant effect (coef = 0.3263, p = 0.025). Other variables such as sex, BMI, alcohol, hypertension, diabetes, CAD, steroid and EFT were not statistically significant predictors in this model (p > 0.05). Please see Table 4.

**Table 4:** Logistic regression model to analyze the predictive value of individual patient characteristics on the dependent variable of Atrial Fibrillation. ISD = Maximum short axis diameter of the interatrial Septum. Non-significant parameters were as follows: Sex, BMI, Alcohol, Hypertension, Diabetes, Coronary Artery Disease, Steroid, Epicardial Fat Tissue.

Variable	coefficient	Std. error	t	P value	95% Confidence
age	0.0476	0.018	2.628	**0.009**	[0.012, 0.083]
tumor	−0.9365	0.287	−3.263	**0.001**	[−1.499, −0.374]
smoking	−0.6447	0.296	−2.179	**0.029**	[−1.224, −0.065]
ISD	0.3263	0.145	2.249	**0.025**	[0.042, 0.611]

## 4 Discussion

This study provides the first volumetric analysis of lipomatous hypertrophy of the interatrial septum (LHIS) in a cohort of 473 patients, examining risk factors, associations with thoracic fat composition, and clinical impact.

The interatrial septal diameter (ISD) measured in the non-anatomical axial CT plane showed a strong correlation with septal volume (Pearson’s r = 0.82), indicating that maximum ISD on routine axial images is a reliable surrogate for volumetric assessment. Thus, additional angulation or dedicated volumetric analysis may not be necessary in standard protocols, where time and resources for volumetric reconstructions may be limited.

The inclusion threshold of ≥ 1 cm ISD was intentionally chosen to capture the full spectrum of interatrial fat accumulation. While a septal thickness > 2 cm is widely accepted as the diagnostic threshold for LHIS [9], [10], inclusion of patients with 1–2 cm thickness allowed assessment of potential clinical associations in earlier stages of fat accumulation. Accordingly, interatrial septal thickness was categorized into < 2 cm, 2–3 cm, and ≥ 3 cm based on established imaging definitions of LHIS. Thicknesses < 2 cm represented subthreshold fat accumulation, 2–3 cm corresponded to classical LHIS, and ≥ 3 cm identified patients with more pronounced septal thickening.

Regression analyses demonstrated significant associations of maximum ISD and septal volume with atrial fibrillation (AF), steroid use, and epicardial fat volume, suggesting a relationship with interatrial fat burden rather than causality. Two large population-based studies support the estimate that cardiac arrhythmias, particularly atrial fibrillation, affect approximately 2–2.5% of the general adult population [24], [25]. AF prevalence in this cohort was 18%, likely reflecting the high mean age (68 years). The probability of AF increases markedly with age, a trend well documented in the literature [26], [27], [28]. This age-related increase is also reflected in our logistic regression analysis, where older individuals showed a significantly higher likelihood of AF. Importantly, we also found a significant association between the maximum ISD of the interatrial septum and atrial fibrillation, even when controlling for age as a covariate. Hence, this study is, to our knowledge, the first large-scale investigation indicating that LHIS may indeed be associated with an increased risk of AF, as suggested by prior case reports. Although the manuscript demonstrates statistical associations between LHIS and atrial fibrillation, causality cannot be established due to the retrospective cross-sectional design, and any statements implying that LHIS reflects a specific metabolic phenotype or predicts atrial fibrillation should be regarded as exploratory and interpreted with particular caution.

The regression analysis identified significant negative coefficients for the variables malignant tumor and smoking, suggesting that individuals with malignant tumors or who smoke were less likely to have AF in this cohort. This unexpected finding may be due to a sampling effect or selection bias related to the indication for CT imaging, as approximately 44% of the scans were performed for staging purposes. This may limit the generalizability of the findings to broader populations, as patients undergoing oncologic staging often differ systematically from the general hospital population with respect to comorbidity burden, prior therapies, and body composition, potentially leading to an over- or underestimation of the reported associations. In addition, the spectrum of disease severity and indications represented in this cohort may not reflect that of patients undergoing thoracic CT in non-oncologic settings, which should be considered when interpreting the validity of the results.

Additionally, male sex and current or prior alcohol consumption emerged as a significant predictor specifically for the maximum ISD and coronary artery disease was linked with the maximum volume of the LHIS. Interestingly, age did not influence maximum ISD and volume of the interatrial septum.

Regarding epicardial and mediastinal fat tissue, (female) sex, higher age, higher BMI, and current or prior steroid medication all demonstrated significant influence, with greater values of these factors corresponding to increased fat tissue volume. Furthermore, the regression revealed a significant negative coefficient for the variable malignant tumor regarding epicardial fat volume, which may reflect a catabolic state or cancer-related metabolic alterations leading to reduced fat tissue. However, since the leading indication for the CT within this sample is tumor staging, this effect is likely driven at least in part by sampling bias and should be interpreted with caution rather than as a protective effect. Similarly, the observed negative associations between smoking and atrial fibrillation, as well as malignant tumor status and atrial fibrillation, are counterintuitive and probably reflect characteristics of this specific oncologic cohort and residual confounding; these findings are therefore considered exploratory and should not be overinterpreted as evidence of a true inverse relationship. While epicardial fat volume increased progressively with ISD size, mediastinal fat volume only differed significantly between group 1 and the other two groups, but not between groups 2 and 3.

This study contributes to a more precise characterization of patients with LHIS, particularly given the lack of data from large patient cohorts to date. As demonstrated by Mallio et al. [20], the coexistence of LHIS and visceral adiposity may reflect a shared metabolic, genetic, and lifestyle background. All of the aforementioned influencing factors represent pieces of a mosaic that may aid in distinguishing a benign from a malignant lesion. In many cases, a (well-differentiated) liposarcoma originating from the interatrial septum was considered as a differential diagnosis of LHIS. However, to the best of our knowledge, in the few cases where histopathological evaluation was performed, no liposarcoma of the interatrial septum could be confirmed [29], [30], [31].

Neither septal thickness nor volume alone reflects the clinical consequences of fat accumulation. In particular, potential hemodynamic effects, such as obstruction of adjacent structures like the superior vena cava, remain unclear and warrant further investigation.

## 5 Limitations

This study has limitations. The study cohort was identified through radiology report keyword searches combined with manual review of thoracic CT examinations over one year, which may introduce selection bias, particularly because a substantial proportion of the included scans were performed for oncologic staging purposes.

The manuscript includes multiple regression analyses; however, several potentially relevant cardiovascular confounders are not sufficiently explored, including dyslipidemia, obstructive sleep apnea, inflammatory disorders, or detailed cardiac functional parameters, and these omissions should be acknowledged more clearly. Moreover, BMI does not provide information about the distribution of fat across individual body compartments. It must also be assumed that not all noxious substances, steroid use, and comorbidities were consistently documented in the hospital information system. A limitation of this study is the use of binary (yes/no) variables for smoking and steroid use rather than quantitative measures. This limits interpretation of the regression analyses and the reported associations may be less dependable. With regard to steroid medication specifically, neither duration nor dosage was recorded. Nicotine and alcohol use were also not quantified in terms of pack-years or consumption levels. The severity of arterial hypertension was likewise not captured, all these variables were only coded in a binary manner (present/absent). Overall, we included factors that appeared logically relevant as potential influencing variables, but not all conceivable confounders or contributing factors were assessed. The segmentation model was trained on a limited number of scans, and performance estimates based on manual corrections of automated segmentations may be overly optimistic. However, as the model was developed only as a support tool with manual correction of all outputs, rather than as a standalone tool, this does not affect the study results.

## 6 Conclusions

This study is the first to conduct a volumetric analysis of lipomatous hypertrophy of the interatrial septum (LHIS) in a large cohort. First, the results suggest that non-anatomical axial measurement of the interatrial septum is a reliable surrogate parameter for estimating septal volume. So there seems to be no need for additional angulation or dedicated volumetric analysis. Second, presence and extent of LHIS were significantly associated with a higher likelihood of atrial fibrillation. Moreover, significant associations between interatrial fat accumulation and factors such as sex, alcohol consumption, coronary artery disease, steroid medication, and epicardial fat volume were found. Taken together, our findings indicate that LHIS is not merely a benign anatomical variant but may be reflective of an underlying metabolic phenotype characterized by increased adiposity, cardiovascular risk factors, cardiac arrhythmias and systemic inflammation.

## CRediT authorship contribution statement

**Christian Stroszczynski:** Supervision, Resources, Project administration. **Simone Hammer:** Writing – review & editing, Investigation. **Okka Wilkea Hamer:** Writing – review & editing, Resources, Project administration, Methodology, Conceptualization. **Lucca Scheuermeyer:** Software, Formal analysis, Data curation. **Quirin Strotzer:** Writing – review & editing, Software, Methodology. **Luca Kronast:** Investigation, Data curation. **Stefanie Meiler:** Writing – original draft, Project administration, Investigation.

## Ethical Statement

All procedures were performed in compliance with relevant laws and institutional guidelines and have been approved by the appropriate institutional committee(s) (Ethics Committee of the University of Regensburg, 21–2718–101).

The privacy rights of human subjects have been observed and that informed consent was obtained for experimentation with human subjects.

Ethical Statement: This study was conducted in accordance with the Declaration of Helsinki and approved by the ethics committee (approval no. 21–2718_1–101).

## Funding

This research did not receive any specific grant from funding agencies in the public, commercial, or not-for-profit sectors.

## Declaration of Competing Interest

The authors declare that they have no known competing financial interests or personal relationships that could have appeared to influence the work reported in this paper.

## Footnotes

Appendix A Supplementary data associated with this article can be found in the online version at doi:10.1016/j.ejro.2026.100801.
