# Supplementary material for: Lipomatous hypertrophy of the interatrial septum: Risk factors, correlation with thoracic fat composition and clinical impact
Source: Eur J Radiol Open. 2026 Jul 28;17:100801. doi: 10.1016/j.ejro.2026.100801 (PMC13449488; doi:10.1016/j.ejro.2026.100801)
Supplement: Supplementary file 1 [file mmc1.docx]

**Supplementary Material**

| patient characteristics | n (%) |
| --- | --- |
| **sex** |  |
| male | 344 (72.7) |
| female | 129 (27.3) |
| **Metabolic disorders, comorbidities, noxes and medication** |  |
| arterial hypertension | 272 (57.5) |
| diabetes | 141 (29.8) |
| coronary artery disease | 97 (20.6) |
| selected cardiac arrhythmias | 113 (23.9) |
| atrial fibrillation | 83 (17.5) |
| tachycardia | 17 (3.6) |
| AV-block | 13 (2.7) |
| nicotine | 217 (45.9) |
| alcohol | 65 (13.7) |
| steroid medication | 189 (40.1) |

**Table S1**, Patient characteristics

| CT indication | n (%) |
| --- | --- |
| tumor/staging | 209 (44.2) |
| pulmonary embolism | 82 (17.3) |
| other | 61 (12.9) |
| known interstitial lung disease/airway disease | 54 (11.4) |
| infectious focus | 35 (7.4) |
| cardiac CT | 12 (2.5) |
| suspected lung disease | 9 (1.9) |
| bleeding | 5 (1.1) |
| postoperative status | 3 (0.6) |
| not available | 3 (0.6) |

**Table S2**, CT indications

| parameter |  | group 1 (n= 269) |  | group 2 (n=163) |  | group 3 (n=41) |  |
| --- | --- | --- | --- | --- | --- | --- | --- |
|  |  | n | % | n | % | n | % |
| **sex** | male | 200/269 | 74.3 | 123/163 | 75.5 | 21/41 | 51.2 |
|  | female | 69/269 | 25.7 | 40/163 | 24.5 | 20/41 | 48.8 |
| **tumor** | yes | 165/269 | 61.3 | 76/163 | 46.6 | 13/41 | 31.7 |
|  | no | 104/269 | 38.7 | 87/163 | 53.4 | 28/41 | 68.3 |
| **smoking** | yes | 108/269 | 40.1 | 83/163 | 50.9 | 26/41 | 63.4 |
|  | no | 161/269 | 59.9 | 80/163 | 49.1 | 15/41 | 36.6 |
| **alcohol** | yes | 30/269 | 11.2 | 27/163 | 16.6 | 8/41 | 19.5 |
|  | no | 239/269 | 88.8 | 136/163 | 83.4 | 33/41 | 80.5 |
| **hypertension** | yes | 143/269 | 53.2 | 102/163 | 62.6 | 27/41 | 65.9 |
|  | no | 126/269 | 46.8 | 61/163 | 37.4 | 14/41 | 34.1 |
| **diabetes** | yes | 72/269 | 26.8 | 54/163 | 33.1 | 15/41 | 36.6 |
|  | no | 197/269 | 73.2 | 109/163 | 66.9 | 26/41 | 63.4 |
| **CAD** | yes | 38/269 | 14.1 | 47/163 | 28.8 | 12/41 | 29.3 |
|  | no | 231/269 | 85.9 | 116/163 | 71.2 | 29/41 | 70.7 |
| **steroid** | yes | 90/269 | 33.5 | 72/163 | 44.2 | 27/41 | 65.9 |
|  | no | 179/269 | 66.5 | 91/163 | 55.8 | 14/41 | 34.1 |
| **AF** | yes | 38/269 | 14.1 | 33/163 | 20.2 | 12/41 | 29.3 |
|  | no | 231/269 | 85.9 | 130/163 | 79.8 | 29/41 | 70.7 |

**Table S3**, Comparison of the ISD-Subgroups – Sex (1 = male, 0 = female), Tumor, Smoking, Alcohol, Hypertension, Diabetes, Coronary Artery Disease (CAD), Atrial Fibrillation (AF) and Steroid.
